# The diagnosis of scabies by non-expert examiners: A study of diagnostic accuracy

**DOI:** 10.1371/journal.pntd.0007635

**Published:** 2019-08-19

**Authors:** Millicent H. Osti, Oliver Sokana, Christina Gorae, Margot J. Whitfeld, Andrew C. Steer, Daniel Engelman

**Affiliations:** 1 Tropical Diseases, Murdoch Children’s Research Institute, Melbourne, VIC, Australia; 2 Department of Paediatrics, University of Melbourne, Melbourne, VIC, Australia; 3 Melbourne Children’s Global Health, Melbourne, VIC, Australia; 4 Ministry of Health and Medical Services, Honiara, Solomon Islands; 5 Department of Dermatology, St Vincent’s Hospital, Sydney, NSW, Australia; 6 School of Medicine, University of New South Wales, Sydney, NSW, Australia; 7 Department of General Medicine, Royal Children’s Hospital, Melbourne, VIC, Australia; QIMR Berghofer Medical Research Institute, AUSTRALIA

## Abstract

**Background:**

Although scabies is estimated to be one of the most common skin conditions globally, prevalence data is not available in most settings. Disease mapping is required to develop and monitor successful control programs. Non-expert health workers are likely to play an important role in scabies mapping activities in endemic settings.

**Methodology:**

Four non-expert health workers were trained in the diagnosis of scabies and impetigo. The health worker diagnosis was compared to a reference consensus diagnosis of two doctors experienced in diagnosis. The study was conducted in a primary school in Gizo, Solomon Islands, in August 2018. The six examiners consecutively assessed school students, blinded to each other’s findings. Training and diagnostic procedures followed criteria for scabies diagnosis established by the International Alliance for the Control of Scabies in 2018.

**Principal findings:**

Amongst the 171 students who underwent clinical assessment the prevalence of scabies and impetigo according to the reference standard was 55% and 45% respectively. Sensitivity of the non-expert health workers’ diagnosis compared to the reference standard was 55.3% for scabies (95% confidence interval [CI], 50.1–60.4) with a specificity of 89.9% (95% CI 86–93.1) and 52.6% for impetigo (95% CI 46.9–58.3) with a specificity 97.8% (95% CI 95.7–99). Sensitivity for moderate to severe scabies was 93.5% (95% CI 86.3–97.6) with a specificity of 74% (95% CI 70.2–77.5).

**Conclusions:**

Following brief training, the diagnostic accuracy of non-expert health workers for scabies and impetigo was promising, especially for moderate to severe disease. Modifications to training and processes are recommended to further improve accuracy. The diagnosis by non-expert health workers may be acceptable for scabies and impetigo mapping in endemic areas.

## Author summary

Scabies is a parasitic infection that leads to significant morbidity worldwide. Mapping of scabies prevalence would improve the understanding of the true burden of disease and the need for control programs in specific countries and regions. The diagnosis of scabies in low resource settings, where the disease is most prevalent, is reliant on clinical examination. A task shifting approach, utilizing local health staff, could substantially increase the feasibility to undertake mapping surveys in low-resource settings. In this study, we aimed to evaluate the diagnostic accuracy of clinical assessment of local health workers following brief training. Our study found that these non-expert health workers could diagnose scabies with moderate accuracy and diagnose more severe disease with high accuracy. Further work is needed to develop standardized training packages to ensure a high level of diagnostic accuracy by non-expert health workers.

## Introduction

Human scabies is a parasitic skin condition caused by infestation with the mite *Sarcoptes scabiei* var. *hominis*. Scabies is a significant public health problem, affecting more than 200 million people globally, with the highest burden in low-resource settings in tropical and subtropical regions [1, 2]. Scabies frequently leads to bacterial skin infection (impetigo), which places individuals at risk of developing serious sequalae including invasive bacterial infections, poststreptococcal glomerulonephritis and possibly rheumatic fever [3–5].

The need for global control of scabies has been recognized by the World Health Organization with the recent addition of scabies to the Department of Neglected Tropical Disease (NTD) Control portfolio [6]. In order to achieve development and implementation of effective control programs, accurate burden estimates are needed. This requires development of an easily implementable protocol for standardized diagnosis in settings where scabies is endemic. Scabies disproportionately affects low resource communities, where clinical diagnosis remains the most commonly used method in programs and research, performed by experts or by local health staff such as nurses [7]. However, clinical diagnostic methods and case definitions are not consistent across studies [7, 8].

Diagnostic criteria, developed through a global Delphi consensus study, and published in 2018 by the International Alliance for the Control of Scabies (IACS), aim to improve the standardization of diagnosis for observational and interventional research [9]. The IACS Criteria enable diagnosis at three levels of diagnostic certainty: level A—Confirmed Scabies; level B—Clinical Scabies; or level C—Suspected Scabies (Table 1). Levels B and C aim to facilitate standardized diagnosis in settings where clinical assessment is the only available or practical diagnostic tool. In the absence of the development of a simple diagnostic test and an expected shortage of available experts, trained non-expert examiners are likely to play a central role in future population screening. This reflects the ‘task shifting’ approach suggested by the World Health Organization in order to maximize human resources by the reallocation of specific tasks to health workers with reduced training time and less qualifications [10]. Brief training of health workers can improve their diagnostic ability following clinical assessment [11]. The clinical assessment of trained, non-expert examiners has been used for diagnosis and mapping for other NTDs including trachoma, leprosy and podoconiosis [12–14].

Although the IACS Criteria provide a consensus approach for scabies diagnosis, they have not yet been validated. The feasibility of implementing these criteria for scabies in a low-resource setting, and the accuracy of non-experts in using the criteria remain unknown. Therefore, we aimed to investigate the diagnostic accuracy of briefly trained, non-expert examiners in the diagnosis of scabies, using the IACS criteria.

## Methods

### Ethics statement

Ethics approval was granted by the Royal Children’s Hospital Melbourne Human Research Ethics Committee (reference number 38099A) and the Solomon Islands Health Research and Ethics Review Board (reference number 05/18).

### Study design and setting

This single site, prospective study of diagnostic accuracy took place in Gizo, a town in the Western Province of the Solomon Islands, with an estimated population of 7,177 [15]. The Solomon Islands is categorized as a low human development country, based on the United Nation’s Human Development Index, ranking 156 out of 188 countries and territories [16]. A survey of scabies in the Western Province (but not including Gizo town) in 2014 estimated the prevalence of scabies to be 19.2% and the prevalence of impetigo to be 32.7% [17]. The study followed the STARD criteria for studies of diagnostic accuracy (S3 Table) [18].

### Participants

The Western Province Ministry of Health recruited four nurses from surrounding towns to travel to Gizo for the study. The nurses provided informed written consent to participate.

Students from a single primary school were consecutively enrolled from August 21 to 24, 2018. Information was sent out to families in the weeks leading up to study. Members from the study team attended a meeting with teachers and parents to answer any questions. Signed written consent was obtained from parents or guardians of all participating students. All students with parental consent were eligible for inclusion. There were no exclusion criteria.

All participants underwent six sequential, blinded clinical assessments (by four non-expert examiners and two expert examiners) at Gizo Primary School over four days. Each child was assessed by all examiners on the same day. Data were collected on skin examination and history features based on the components of the IACS Criteria.

### Training

Four nurses were recruited to participate based on availability and pre-defined desirable attributes and skills, similar to the indicators used by the Global Trachoma Mapping Project (GTMP) [13]. These included effective communication skills, ability to work long days, capacity to work with technological devices and ability to travel for fieldwork. The nurses were current employees of the Ministry of Health working in local health centers clinics or hospitals. They were aware of scabies and impetigo through previous professional experience but had no formal training in diagnosis of these skin conditions prior to the study. In August 2018, the nurses participated in an intensive two-day training program led by two doctors with extensive experience in the clinical diagnosis of scabies and in training of skin disease. A third doctor provided background information on the public health importance of scabies. Training consisted of interactive tutorials focused on the clinical features of scabies, based on the IACS Criteria [9]. At the time of the study, only the basic criteria levels were published, and detailed information on the criteria (for example, how to define ‘typical lesions’ and ‘typical distribution’) was not available. Trainers therefore interpreted these terms using previous experience. Nurses were also trained in the diagnosis of bacterial skin infection, which frequently complicates scabies lesions (causing infected scabies), or can coexist as impetigo [19]. They were also trained to identify other locally relevant skin conditions that may present similarly to scabies.

**Table 1:** IACS Criteria for the diagnosis scabies.

**A:**	**Confirmed scabies**
A1:	Mites, eggs or feces on light microscopy of skin samples
A2:	Mites, eggs or feces visualized on individual using high powered imaging device
A3:	Mite visualized on individual using dermoscopy
**B:**	**Clinical Scabies**
B1:	Scabies burrows
B2:	Typical lesions affecting male genitalia
B3:	Typical lesions in a typical distribution and two history features
**C:**	**Suspected Scabies**
C1:	Typical lesions in a typical distribution and one history feature
C2:	Atypical lesions or an atypical distribution and two history features
	**History Features**
H1:	Itch
H2:	Close contact with an individual who has itch or typical lesions in a typical distribution

A diagnosis of scabies should only be made if other differential diagnoses are considered less likely than scabies.

### Written assessment

As a component of training, the nurses took a written test consisting of 50 clinical cases, similar to the GTMP grader training slide-test [13]. Each case included one clinical photograph and relevant history features. Nurses were instructed to indicate the presence or absence of scabies and/or bacterial skin infection. Responses were marked against the consensus diagnosis of two experts. There was no minimum competency required in order to progress to the clinical assessment.

### Clinical assessment

Supervised, practical training was then conducted in the school setting and included participants with and without the target skin conditions. Diagnosis relied on clinical assessment (IACS Criteria Level B, Clinical Scabies, and C, Suspected Scabies, Table 1). Neither microscopy nor dermoscopy were used by any examiner for reasons of logistics and cost, and to resemble the conditions that may be anticipated as part of a scabies mapping surveys in a low-resource setting. There was no specified time limit for clinical examination.

The clinical assessment was a focused skin examination of exposed areas. The children wore school uniforms consisting of shorts and a short-sleeved shirt or a short-sleeved school dress. Shoes (most often sandals) were removed prior to assessment. Therefore, exposed areas were neck, face and scalp, from fingers to upper arms, and from toes to upper thighs. The genitals were not examined so the IACS subcategory B2 (typical lesions affecting male genitalia) was not included.

For the purpose of this study, typical lesions were defined as multiple small lumps (papules, nodules or vesicles) occurring in the same area. Typical distribution of lesions was defined as lesions on the fingers, finger-webs, wrists, hands, forearms toe-webs, feet and legs. All examiners were provided with a body diagram outlining the regions considered typical for scabies lesions. Scabies severity was defined by number of lesions, consistent with previous studies [17, 20]: mild, 1–10 lesions; moderate, 11–49 lesions; or severe, ≥ 50 lesions.

Each examiner also assessed the two history components of the IACS Criteria (presence of itch, and contact history). A positive response to any of the four following questions was considered as a positive contact history: whether an individual lived with someone with itch; lived with someone with a rash that looked like scabies; had a friend or classmate with itch; or had a friend or classmate with a rash that looked like scabies. Students were shown reference photographs of typical scabies rashes to aide these questions.

Impetigo was defined as papules, pustules or ulcerative lesions with associated erythema, crusting or pustular exudate. Impetigo included both infected scabies lesions and discrete impetigo lesions. Severity of impetigo was defined as: very mild (1–5 lesions); mild (6–10 lesions); moderate (11–49 lesions); or severe (≥ 50 lesions) [21].

### Index test

The index test was the clinical diagnosis of scabies (and impetigo) by non-expert examiners (the four nurses) following training.

### Reference standard

The reference standard was the diagnosis made by two medical doctors (a dermatologist and a pediatrician) with extensive experience in the diagnosis of scabies and tropical skin diseases. The two doctors independently assessed participants for scabies (using the IACS Criteria) and bacterial skin infection. Where there was disagreement on the presence of scabies or impetigo, the participant was re-examined by both doctors together to establish a consensus diagnosis.

### Analysis

Sample size was calculated based on an anticipated sensitivity and specificity of 90% for scabies diagnosis, with a confidence interval (CI) of 80–99%. This required enrolment of at least 40 individuals with scabies and 40 without scabies [22]. However, the school principal requested that the study team examine all individuals who provided consent. Therefore, as many participants as feasible were enrolled within the four-day period when all six examiners were available. Eligible participants that could not be examined during this time were examined off-study at a later date.

Sensitivity and specificity were calculated for the diagnosis of scabies and impetigo by comparing the index test to the reference standard. As secondary outcomes, the accuracy in diagnosis of more severe forms of each disease was also calculated. Agreement between the two experts on the diagnosis of scabies and impetigo was calculated using Cohen’s kappa coefficient [23]. Inter-rater agreement for the reporting of itch and contact history was calculated using Fleiss’s kappa, for all participants where an answer was recorded by all six examiners [24]. Stata 15.1 (Statacorp, College Station, TX, USA) was used for all statistical calculations.

### Treatment

All participants diagnosed with scabies by expert examiners, as well as their household contacts, were offered treatment with permethrin 5% cream. Individuals diagnosed with mild impetigo were provided information on home-based management. Individuals with moderate or severe impetigo, or other conditions requiring treatment, were referred to the local health service.

## Results

**Fig 1:**
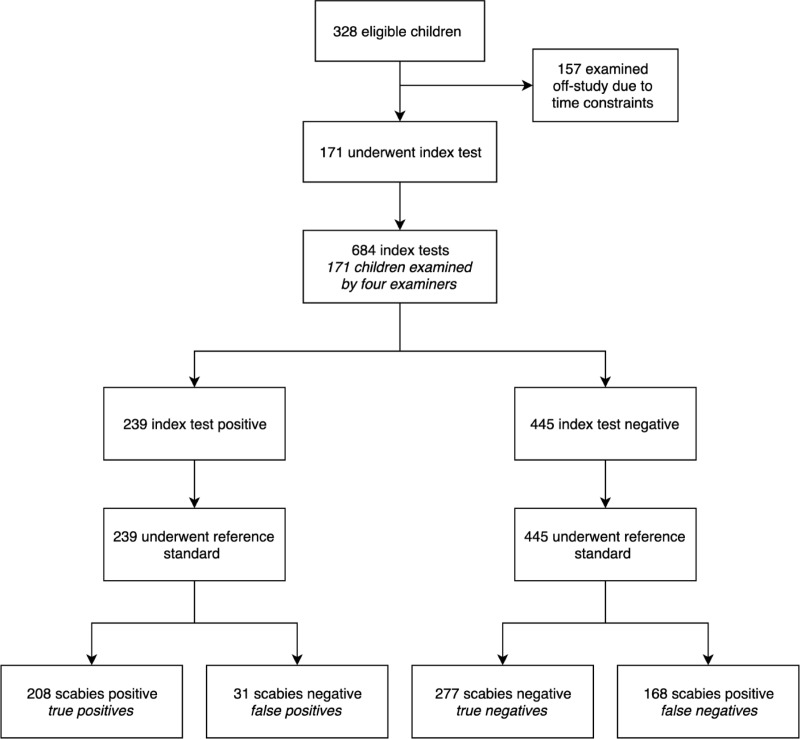
Flow of participants for the diagnosis of scabies.

171 individuals participated, and all were assessed by four non-expert examiners and two experienced doctors, resulting in a paired index and reference test (Fig 1). There were no adverse events. The mean age of the children was 8.8 years (standard deviation 2.3, range 4–14) and 51.5% were female (Table 2).

**Table 2:** Severity of disease and clinical characteristics.

Characteristic	N = 171	%
**Scabies**	**n = 94**	55.0
Severity		
Mild (1–10 lesions)	63	67.0
Moderate (11–49 lesions)	21	22.3
Severe (≥50 lesions)	10	10.6
IACS Criteria		
Clinical Scabies		
B1: Scabies Burrows*	4	2.3
B3: Typical lesions in a typical distribution with itch and positive contact	65	69.1
Suspected Scabies		
C1: Typical lesions in a typical distribution with itch or positive contact	23	24.5
C2: Atypical lesions or typical distribution with itch and positive contact	6	6.4
**Impetigo**	**n = 77**	45.0
Severity		
Very mild (1–5 lesions)	64	83.1
Mild (6–10 lesions)	8	10.4
Moderate (11–49 lesions)	2	2.6
Severe (≥50 lesions)	3	3.9

*Individuals with burrows were additionally categorized as either B3, C1, C2

**Fig 2:**
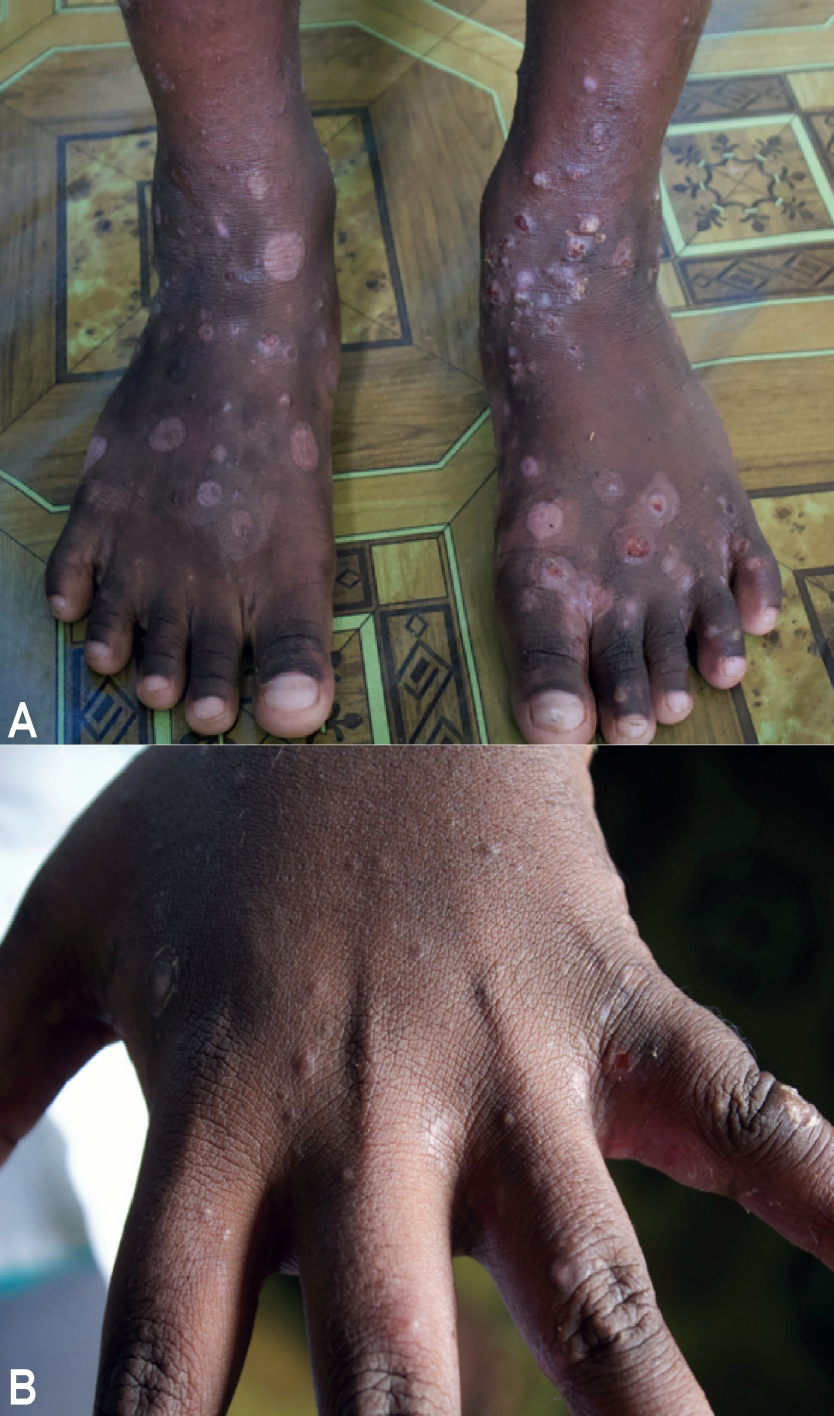
Example appearance of scabies lesions (A. Scabies lesions over feet and ankles with bacterial super-infection, B. Papular scabies lesions over fingers, finger-webs and dorsum of hand).

According to the reference standard, the prevalence of scabies was 55% (95% CI 47.2–62.6), and 67% of these had mild disease (Table 2). The prevalence of impetigo was 45% (95% CI 37.4–52.8), and 83.1% of these had very mild disease (Table 2). Of individuals with scabies, 47 (50.5%) had bacterial superinfection or co-infection (Fig 2).

The prevalence of scabies according to the two individual doctors was 53.2% (95%CI 45.4–60.9) and 49.7% (95%CI 42–57.4) respectively.

**Fig 3:**
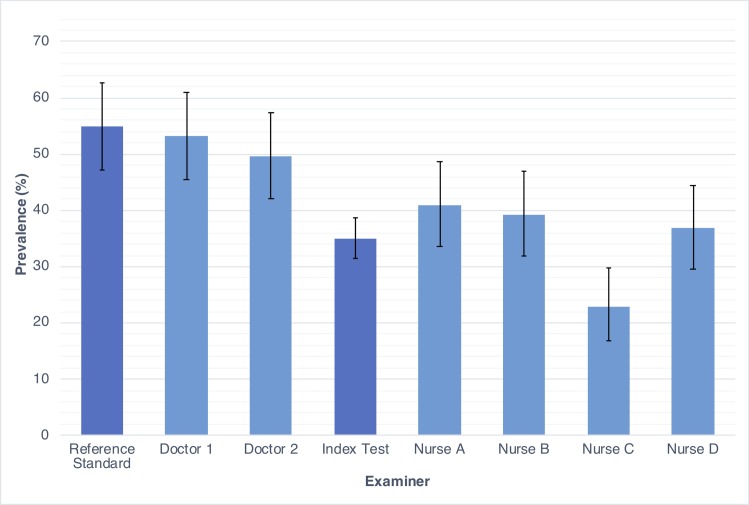
Prevalence of scabies by examiner with 95% confidence intervals.

Overall, the prevalence of scabies according to the index test (briefly trained nurses) was 34.9% (95% CI 31.4–38.6, Table 3). Prevalence by individual examiner ranged from 22.8% to 40.9% (Fig 3). The prevalence of impetigo according to the index test was 25.2% (95% CI 21.9–28.7, range of individual examiners 18.7% to 32.2%, Table 4).

**Table 3:** Accuracy of scabies diagnosis.

	Participants Screened (n)					Scabies % (95%CI)	Measurement of accuracy (95%CI)				
	TP	FP	FN	TN	Total	Prevalence	Sensitivity	Specificity	PPV	NPV	OR
Nurse A	60	10	34	67	171	40.9 (33.5–48.7)	63.8 (53.3–73.5)	87 (77.4–93.6)	85.7 (73.3–92.9)	66.3 (56.2–75.4)	11.8 (5.4–25.7)
Nurse B	61	6	33	71	171	39.2 (31.8–46.9)	64.9 (54.4–74.5)	92.2 (83.8–97.1)	91 (81.5–96.6)	68.3 (58.4–77.1)	21.9 (8.8–54.3)
Nurse C	39	0	55	77	171	22.8 (16.8–29.8)	41.5 (31.4–52.1)	100 (95.3–100)	100 (91–100)	58.3 (49.4–66.8)	*
Nurse D	48	15	46	62	171	36.8 29.6–44.5)	51.1 (40.5–61.5)	80.5 (69.9–88.7)	76.2 (63.8–86)	57.4 (47.5–66.9)	4.31 (2.2–8.6)
Total	208	31	168	277	684	34.9 (31.4–38.6)	55.3 (50.1–60.4)	89.9 (86–93.1)	87 (82.1–91)	62.2 (57.6–66.8)	11.1 (7.3–16.9)

TP = true positive, FP = false positive, FN = false negative, TN = true negative

*OR could not be calculated due to 0 values

**Table 4:** Accuracy of impetigo diagnosis.

	Participants Screened (n)					Impetigo % (95%CI)	Measurement of accuracy (95%CI)				
	TP	FP	FN	TN	Total	Prevalence	Sensitivity	Specificity	PPV	NPV	OR
Nurse A	36	1	39	84	160	23.1 (16.8–30.4)	48.0 (36.3–59.8)	98.8 (93.6–100)	97.3 (85.8–99.9)	69.3 (59.3–76.4)	77.5 (12.9-)
Nurse B	42	3	35	89	169	26.6 (20.1–34)	54.5 (42.8–65.9)	96.7 (90.8–99.3)	93.3 (81.7–98.6)	71.8 (63–79.5)	35.6 (10.9–115)
Nurse C	53	2	24	92	171	32.2 (25.2–39.7)	68.8 (57.3–78.9)	97.9 (92.5–99.7)	96.4 (87.5–99.6)	79.3 (70.8–86.3)	102 (25.3-)
Nurse D	30	2	47	92	171	18.7 (13.2–25.4)	39.0 (28.0–50.8)	97.9 (92.5–99.7)	93.8 (79.2–99.2)	66.2 (57.7–74)	29.4 (7.4-)
Total	161	8	145	357	671	25.2 (21.9–28.7)	52.6 (46.9–58.3)	97.8 (95.7–99)	95.3 (90.9–97.9)	71.1 (66.9–75)	49.5 (24.1–102)

TP = true positive, FP = false positive, FN = false negative, TN = true negative

The sensitivity of the index test compared to the reference standard for scabies diagnosis was 55.3% (95% CI 50.1–60.4, Table 3). Sensitivity for individual non-expert examiners ranged from 41.5% to 64.9% (Table 3). The specificity of the index test was 89.9% (95% CI 86–93.1, range of individual examiners 87.0% to 100%). When considering only the moderate and severe cases of scabies (n = 31), the sensitivity of the index test was 93.5% (95% CI 86.3–97.6, range of individual non-expert examiners 87% to 95.7%). The specificity of the index test for moderate and severe scabies was 74.0% (95% CI 70.2–77.5, range of individual non-expert examiners ranged 67.3% to 87.1%, S1 Table).

For impetigo, the sensitivity of the index test was 52.6% (95% CI 46.9–58.3, range of individual non-expert examiners 39.0% to 68.8%), and specificity was 97.8% (95% CI 95.7–99, range of individual non-expert examiners 96.7% to 98.8%, Table 4). If only cases of impetigo with six or more infected lesions were considered (n = 13), then the sensitivity of the non-expert nurses was 84.6% (95% CI 71.9–93.1) and the specificity was 79.8% (95% CI 76.4–82.9).

Repeat assessment was required to define the reference standard for 46 out of 171 (26.9%) cases due to disagreement on presence of scabies or impetigo between the doctors. Agreement between the two experts for scabies was 83.6% (kappa = 0.67) and for moderate to severe scabies was 91.3% (kappa = 0.68). Agreement between the experts for impetigo was 87.1% (kappa = 0.73).

The inter-rater agreement (Fleiss kappa) for the reporting of history by individuals between the six examiners was 0.63 for self-reported itch and 0.52 for any contact history (S2 Table).

The results of the written test completed during training for each non-expert examiner were 76%, 92%, 96% and 96% correct for scabies respectively, and 70%, 76%, 76% and 80% correct for impetigo respectively.

## Discussion

We found that briefly trained, non-expert examiners had moderate sensitivity and high specificity in the diagnosis of scabies using the IACS Criteria, compared to reference diagnosis by two expert examiners. The accuracy of examiners is dependent on the effectiveness of training and examination protocols. Our results suggest that modifications to the training protocol are needed to maximize the accuracy and utility for non-expert scabies assessment. More detailed information regarding the IACS Criteria, particularly relating to the definition of typical lesions, is expected to be available soon, which will inform components of training [9].

Although a sensitivity of 55% for scabies diagnosis is lower than expected, a sensitivity of 94% for mild to moderate cases is substantial. Our results are similar to a previous study in Fiji, where nurse examiners’ diagnosis was compared to a single experienced examiner, with diagnosis based on the Integrated Management of Childhood Illness protocol [25]. Nurses in that study had a higher sensitivity (89%) for cases of infected scabies (which may be representative of more severe disease) compared to non-infected scabies (58%). In our study, 66% of scabies cases were mild, similar to the severity found in other studies [17, 20]. We suggest future training incorporate a more specific focus on the diagnosis of mild scabies.

The lower sensitivity in the diagnosis of mild disease may have reflected the normalization of mild cases by nurses, and strict diagnosis by expert examiners. This is supported by the higher prevalence of scabies and impetigo by the reference standard (55% and 45% respectively) compared to the index test (35% for scabies and 25% for impetigo). Scabies and impetigo can be under-recognized by local health care workers at the health clinic level [26], and therefore mapping of scabies and impetigo using non-expert examiners may under-report the true disease prevalence. Improved awareness of scabies in communities and amongst health care workers where it is endemic, as well as the suggested modifications to training, may lead to improvements in accuracy.

Sensitivity for scabies diagnosis varied between the nurse examiners, from 41% to 65%, despite the same training and similar background experience. There were few false positives, reflected by the narrow specificity range among nurses (87–100%). This again highlights that missed mild disease was the major issue encountered. The range in accuracy is as expected and illustrates the inherent variability in proficiency among health care workers at all levels. If examiners were to be employed for mapping exercises, a minimum competency (for example, of ≥ 80%) should be achieved for accreditation, similar to methods used in training for trachoma [13] and other diseases.

The written, picture-based test was a valuable component of the training, enabling trainers and nurses to identify areas for further training, prior to commencing supervised practical training. The written test allowed trainers to revisit concepts that individuals may have not grasped adequately, similar to the role of the GTMP slide-assessment [13]. We propose this be used as a hurdle-requirement during training that must be passed to proceed to practical training. Refinements to the written test, such as the inclusion of more mild cases, may ensure that the test is more representative of the clinical disease profile.

This study was the first to implement the IACS Criteria for scabies diagnosis. These criteria were easily adapted for data collection, allowing standardization of diagnosis amongst the examiners. However, the criteria rely on self-reported itch and contact history, which may be less reliable components. Itch was reported more consistently by the participants (Fleiss kappa = 0.63) than contact history (Fleiss kappa = 0.52). Potentially, these self-reported history components may be less reliable in children than in adults.

We found a very high prevalence of scabies (55%) amongst the participants in the study. This is much higher than previous figures reported by the Solomon Islands Ministry of Health. It is likely that this high burden of disease has a detrimental impact on schooling and health outcomes, highlighting the need for further epidemiologic mapping and control responses in these settings.

This study has several limitations. First, all evaluations of scabies diagnosis are limited by the lack of an objective reference or “gold” standard for diagnosis. However, agreement between the expert examiners was good (kappa = 0.67 for scabies, kappa = 0.73 for impetigo). Second, the blinding of expert assessment and the requirement of consensus diagnosis between two expert examiners was used for a more rigorous diagnostic process. However, it is possible that the consensus process led to an overly sensitive reference standard, particularly as 27% of individuals required re-assessment and discussion for consensus. Many of these were mild cases with a very small number of lesions, where one examiner had thought the most likely cause to be scabies. The potential overdiagnosis may have reduced the diagnostic accuracy of the index test. Third, we only included clinical assessment of school-aged children, and accuracy may be different in other age groups. However, scabies and impetigo present similarly among older children and adults [27], and the inclusion of a wider age group may not yield vastly different results. Additionally, the inclusion of only four non-expert examiners is a limitation, however, this was a feasible number for training and ensured that training was closely supervised. Finally, due to resources and infrastructure available in the school buildings, particularly a lack of electrical power, and conditions of severe heat and humidity, examiners had suboptimal lighting and examination conditions, which may have influenced the results. Although limiting, this environment may reflect the real-world implementation of the index test in settings such as the Solomon Islands. Conditions for examinations in low resource settings may be optimized by the use of torches or external lighting for examination and by emphasizing the need for appropriate working environments.

The results of this study reflect the training program, the participating examiners and the population and may not be generalizable to other settings, particularly those with a lower prevalence. However, this study is an important step toward standardization of diagnosis of scabies and impetigo for mapping and research in low-resource settings. In order for scabies prevalence mapping to occur, either in isolation, or integrated with other NTD assessments, governments and organizations need to be able to feasibly and confidently train local staff in diagnosis. Further refinements to the training and assessment processes are required and may lead to improvements in diagnostic accuracy.

## Supporting information

S1 Table Accuracy of moderate to severe scabies diagnosis. (PDF)

S2 Table Consistency in the reporting of history features. (PDF)

S3 Table STARD checklist. (PDF)
