# Supplementary material for: The diagnosis of scabies by non-expert examiners: A study of diagnostic accuracy
Source: PLoS Negl Trop Dis. 2019 Aug 19;13(8):e0007635. doi: 10.1371/journal.pntd.0007635 (PMC6715246; doi:10.1371/journal.pntd.0007635)
Supplement: S1 Table — (PDF) [file pntd.0007635.s001.pdf]

S1 Table: Accuracy of moderate to severe scabies diagnosis

	Participants Screened (n)					Measurement of accuracy (95%CI)				
	TP	FP	FN	TN	Total	Sensitivity	Specificity	PPV	NPV	OR
Nurse A	29	41	2	99	171	93.5 (78.6-99.2)	70.7 (62.4-78.1)	41.4 (29.8-53.8)	98.0 (93-99.8)	35.0 (8.78-)
Nurse B	30	37	1	103	171	96.8 (83.3-99.9)	73.6 (65.5-80.7)	44.8 (32.6-57.4)	99.0 (94.8-100.0)	83.5 (13.8-)
Nurse C	25	14	6	126	171	80.6 (62.5-92.5)	90 (83.8-94.4)	64.1 (47.2-78.8)	95.5 (90.4-98.3)	37.5 (13.4-105)
Nurse D	28	35	3	105	171	90.3 (74.2-98.0)	75.0 (67.0-81.9)	44.4 (31.9-57.5)	97.2 (92.1-99.4)	28 (8.47-91.5)
Total	112	127	12	433	684	90.3 (83.7-94.9)	77.3 (73.6-80.7)	46.9 (40.4-53.4)	97.3 (95.3-98.6)	31.8 (17.1-59.1)

TP = true positive, FP = false positive, FN = false negative, TN = true negative
