# Supplementary material for: The diagnosis of scabies by non-expert examiners: A study of diagnostic accuracy
Source: PLoS Negl Trop Dis. 2019 Aug 19;13(8):e0007635. doi: 10.1371/journal.pntd.0007635 (PMC6715246; doi:10.1371/journal.pntd.0007635)
Supplement: S2 Table — (PDF) [file pntd.0007635.s002.pdf]

S2 Table: Consistency in the reporting of history features

	N	Agreement %	Fleiss' Kappa	95% CI
**Itch**				
Self-reported itch	83	82.8	0.63	0.52-0.75
**Contact history**				
Household contact with itch	80	81.1	0.60	0.50-0.71
School contact with itch	79	73.8	0.43	0.31-0.55
Household contact with scabies rash	79	82.5	0.59	0.48-0.70
School contact with scabies rash	80	76.8	0.50	0.38-0.61
Any positive contact history*	81	84.0	0.52	0.37-0.67

*Answered yes to any contact history questions
